# Comparative efficacy of glimepiride and metformin in monotherapy of type 2 diabetes mellitus: meta-analysis of randomized controlled trials

**DOI:** 10.1186/1758-5996-5-70

**Published:** 2013-11-14

**Authors:** Hongmei Zhu, Shuang Zhu, Xiuqian Zhang, Yang Guo, Yunzhen Shi, Zhimin Chen, Siu-wai Leung

**Affiliations:** 1 State Key Laboratory of Quality Research in Chinese Medicine, Institute of Chinese Medical Sciences, University of Macau, Macao, China; 2 School of Informatics, University of Edinburgh, Edinburgh EH8 9AB, UK

**Keywords:** Glimepiride, Metformin, Type 2 diabetes mellitus, Meta-analysis

## Abstract

**Background:**

Metformin treatment has been the most recommended monotherapy of type 2 diabetes mellitus (T2DM) for decades but is challenged by new antidiabetic drugs. This study conducted a meta-analysis of randomized controlled trials (RCT) comparing the efficacy of metformin and glimepiride in monotherapy of T2DM.

**Methods:**

A literature search for RCTs on glimepiride and metformin was conducted on the bibliographic databases, including PubMed, Cochrane Library and ScienceDirect, from their inceptions to 25 Mar 2013. All RCTs were selected according to pre-specified eligibility criteria. The quality of articles was assessed with the Cochrane’s risk of bias tool. Statistical meta-analysis evaluated the overall effects and biochemical indices of T2DM. Sensitivity and subgroup analyses evaluated the robustness and explained the heterogeneity of the results. Begg and Egger’s tests quantified possible publication biases. Results were represented as "standard mean difference or odds ratio [95% confidence internals] P value".

**Results:**

Fifteen RCTs with 1681 adult T2DM patients were included for meta-analysis. Metformin was not better than glimepiride in overall efficacy in controlling the levels of HbA1c, postprandial blood sugar (PPBS), fasting plasma insulin (FINS), systolic and diastolic blood pressures (SBP and DBP), and high density lipoprotein (HDL). Metformin was only more effective than glimepiride in controlling the levels of total cholesterol (TC, 0.33 [0.03, 0.63], P = 0.03), low-density lipoprotein (LDL, 0.35 [0.16, 0.53], P = 0.0002) and triglycerides (TG, 0.26 [0.05, 0.46], P = 0.01). Odds ratios of adverse events showed that glimepiride was more likely to induce hypoglycemia episodes and metformin was with a higher risk of gastrointestinal upset.

**Conclusion:**

Metformin was not significantly better than glimepiride in glycemic control of T2DM, suggesting that glimepiride would be a good choice second to metformin in the monotherapy of T2DM.

## Introduction

Metformin has been the most recommended monotherapy of type 2 diabetes mellitus (T2DM) [1,2]. The UK Prospective Diabetes Study (UKPDS) found metformin more effective than chlorpropamide, glibenclamide and insulin [3-5]. The American Diabetes Association (ADA) recommended metformin as the first drug of choice for treating T2DM patients, especially those who are overweight [2]. The UK National Institute for Health and Clinical Excellence (NICE) recommended metformin if the patients are at danger under hypoglycaemia [1]. The latest recommendations of ADA [2] and NICE [1] were updated with the results of UKPDS [6,7], post-trial monitoring of UKPDS [8], and systematic reviews of comparing metformin with placebo, sulfonylureas and other anti-diabetic drugs [7,9], as well as the randomized controlled trials (RCTs) comparing metformin monotherapy with pioglitazone [10], metformin plus nateglinide [11], metformin plus rosiglitazone [12] and other non-metformin treatments [13]. A meta-analysis of RCTs on the efficacy of metformin in treating T2DM [14] found metformin lacking clear evidence for efficacy over the conventional or placebo treatment. A recent literature review suggested that metformin, albeit old, remained the best treatment for T2DM [15] but the review was not a systematic review or meta-analysis. It did not include the latest RCTs comparing metformin and glimepiride in monotherapy of T2DM.

Glimepiride is of the latest generation sulfonylureas for treating T2DM [16]. It has a lower cardiovascular risk than conventional sulfonylureas do [17-19]. Recent RCTs found it comparable to metformin in treating T2DM patients [20,21] including those who are not responding well to non-glimepiride sulfonylureas [22,23]. Probably due to the late launch of glimepiride [24,25] and lack of head-to-head comparative RCTs, early UKPDS, ADA and NICE’s recommendations did not include the results of RCTs comparing metformin with glimepiride in monotherapy but they did include the findings that sulfonlyureas had increased risks in hypoglycemia, weight gain and cardiovascular issues. Recent cohort studies confirmed the increased cardiovascular risks of glimepiride [26] but did no cardiovascular harm to the patients with diagnosed coronary artery disease [27]. This study aimed to compare the efficacy between metformin and glimepiride in monotherapy of T2DM through a meta-analysis and supply the evidence that was missing from previous reviews [9,11] and clinical guidelines [1,2].

## Methods

### Eligibility criteria

#### Inclusion criteria

This study included the RCTs comparing glimepiride with metformin as monotherapy of T2DM. Participants in the RCTs were adult patients suffering from T2DM. Outcome measures of the treatment of T2DM included BMI (body mass index), SBP (systolic blood pressure), DBP (diastolic blood pressure), FPG (fasting plasma glucose), HbA1c (glycosylated hemoglobin level), PPBS (postprandial blood sugar), TC (total cholesterol), HDL (high-density lipoprotein), LDL (low-density lipoprotein), TG (triglycerides) and FINS (fasting plasma insulin).

#### Exclusion criteria

This study excluded the RCTs with the participants who were non-responders to metformin or glimepiride and received dosages exceeded the upper recommended limit (metformin: 2550 mg daily; glimepiride: 8 mg daily) [28]. The RCTs of extremely small sample size (fewer than 10 patients) were also excluded.

### Search and selection of studies

Bibliographical databases, including PubMed, Cochrance Library, Science Direct, China Academic Journals Web Publishing Database, China Master Theses Full-text Database and China Doctor Theses Full-text Database on the China National Knowledge Infrastructure (CNKI), WanFang Data and Google, were searched from their inceptions to 25 Mar 2013.

Search strategies were specified in the working languages of databases, although the terminologies in searching Chinese and English databases were equivalent. Basically, the articles with the terms "glimepiride" and "metformin" in titles, abstracts, and keywords were retriered. Specific search strategies were:

**PubMed:** "Glimepiride" and "Metformin" in Abstract or Title; **Cochrane Llibrary:** "Glimepiride" and "Metformin" in Title, Abstract or Keywords; **Science Direct:** "Glimepiride" and "Metformin" in Abstract, Title, or Keywords; **China Academic Jounrnals Web Publishing Database, China Doctor Theses Full-text Database** and **China Master Theses Full-text Database:** 'Title = Glimepiride * Metformin or Keyword = Glimepiride * Metformin or Abstract = Glimepiride * Metformin’ (in Chinese); **Wan Fang: '**Title All "Glimepiride Metformin" or Keywords All "Glimepiride Metformin" or Abstract All "Glimepiride Metformin"’ (in Chinese).

Two groups of reviewers (three reviewers in each group) independently performed the literature search and selection. The results from one group were cross-checked by the other group. Disagreements were resolved by group discussion.

### Data extraction and quality assessment

Two reviewers (HZ, XZ) independently extracted data of study characteristics and outcome measures from the selected RCTs. The extracted data were cross-checked before quality assessment according to the Cochrane’s risks of bias tool [29]. Disagreement was resolved by discussion between the reviewers (HZ, XZ). A third reviewer (SZ) was consulted when necessary.

### Meta-analysis

Extracted data were transferred to Review Manager 5.2 [30] for meta-analysis with random-effects model. Numeric outcome measures were represented in standardized mean differences (SMD) or odds ratios (OR) and their 95% confidence intervals (CI). Study heterogeneity was evaluated with T^2^ test and I^2^ statistics. P values below 0.05 were considered statistically significant.

### Sensitivity and subgroup analysis

Sensitivity analysis of the efficacy was performed on the sample sizes, on whether the patients received prior anti-diabetic treatments and on the daily dose of metformin. Subgroup analysis was performed to explain the heterogeneity in terms of differences in follow-up periods and characteristics of the participants.

### Adverse events analysis

Adverse events analysis was performed on the hypoglycemia, gastrointestinal upset and overall side effects with their odds ratios (OR) and 95% CI.

### Publication bias

Funnel plots were generated to visualize possible publication bias. Begg and Egger’s tests using the package "metafor" [31] with statistical software R [32] evaluated the statistical significance of the publication bias.

## Results

### Included studies and their characteristics

**Figure 1:**
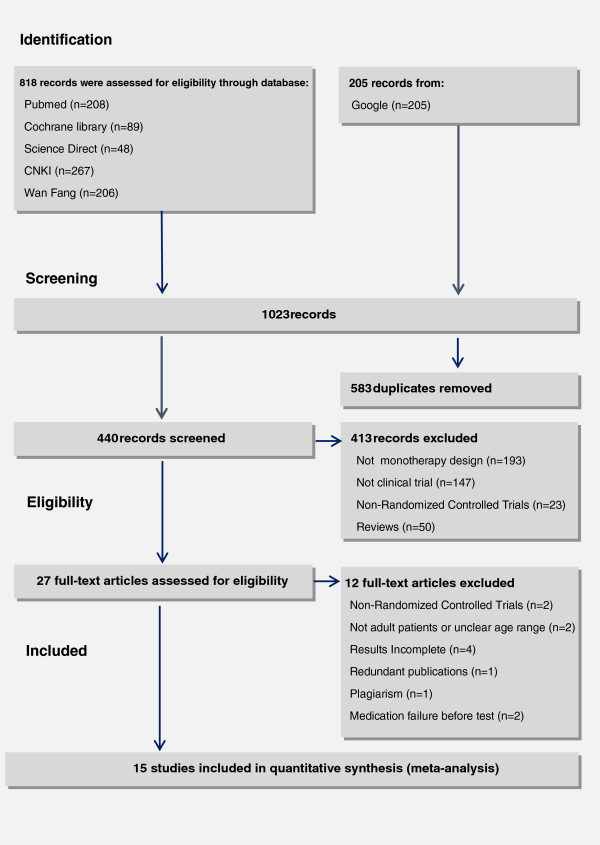
Flow of study selection.

Figure 1 shows the selection process of the studies. A total of 1023 records were identified in accordance with the search strategies from specific bibliographical databases, i.e. PubMed (n = 208), Cochrance Library (n = 89), Science Direct (n = 48), Chinese National Knowledge Infrastructure (n = 267), WangFang (n = 206) and Google (n = 205). Among the 440 records after removal of duplicates, 27 records met the eligibility criteria. After full-text assessment, 12 of 27 studies were excluded for the reasons stated in Figure 1. As a result, 15 RCTs with 1681 participants were included for meta-analysis. The characteristics of the included studies are shown in Table 1.

**Table 1:** Characteristics of included studies

**Author (year)**	**Country**	Age*	**Sample size**		**Dosage**		**Follow-up period (weeks)**	
			**Glimepiride**	**Metformin**	**Glimepiride**	**Metformin**		**Ref**
Ling (2003)	China	Female 54 (8.3)	50	44	1-6 mg/day	750-1750 mg/day	12	[33]
		Male 53 (9.2)						
Ramachandran (2004)	India	30-60	18	21	1-2 mg/day	250-850 mg/day	14	[34]
Derosa (2004)	Italian	46-67	81	83	2-4 mg/day	2000-3000 mg/day	52	[35]
Gonzolez (2004)	Mexico	40-65	37	33	2 mg/day	2000 mg/day	12	[36]
Rong (2004)	China	42 (11)	98	100	4 mg/day	1500 mg/day	12	[22]
Tang (2004)	China	35-70	33	29	1-2 mg/day	750-1500 mg/day	26	[37]
Yamanouchi (2005)	Japan	Metformin 54.7 (9.8)	37	39	1-2 mg/day	750 mg/day	52	[20]
		Glimepiride 53 (9.2)						
Ning (2006)	China	35-70	51	50	1-6 mg/day	250 mg bid-750 mg tid	52	[38]
Wu (2007)	China	30-70	40	30	1-6 mg/day	250-2000 mg tid	12	[39]
Xu (2007)	China	35-70	34	34	1-6 mg/day	250 mg to maximum bid	-	[40]
Li (2007)	China	32-70	35	33	2-6 mg/day	250-1500 mg bid	65	[41]
Wang (2009)	China	45 (7)	49	50	4-6 mg/day	1500 mg/day	12	[23]
Rahman (2011)	Pakistan	Metformin 51.9 (14.1)	102	102	2-8 mg/day	500-2000 mg/day	52	[42]
		Glimepiride 52 (15.4)						
Yoon (2011)	Korea	30-65	118	114	2 mg/day	500 mg/day	48	[21]
Wang (2011)	China	33-70	68	68	2 mg/day	500 mg bid	12	[43]

* presented in range or mean (standard deviation).

### Overall effects

Meta-analysis was performed on the outcome measures FPG, BMI, HbA1c, PPBS, TC, FINS, HDL, LDL, TG, SBP and DBP. The SMD, 95% CI and P values for outcomes between metformin and glimepiride are shown in Table 2. The SMDs between metformin and glimepiride were only statistically significant on TC (0.33 [0.03, 0.63], P = 0.03), LDL (0.35 [0.16, 0.53], P = 0.00002), and TG (0.26 [0.05, 0.46], P = 0.01), indicating that efficacy of metformin was statistically significant over glimepiride in lipid metabolism indices. The differences in glycemic control (e.g. HbA1c and PPBS) and cardiovascular indices (e.g. blood pressure) were not statistically significant. As shown in Table 2, there were significant heterogeneities among studies in SBP (I^2^ = 86%, P < 0.0001), DBP (I^2^ = 87%, P < 0.00001), PPBS (I^2^ = 81%, P < 0.00001), TC (I^2^ = 79%, P < 0.0001), HDL (I^2^ = 86%, P < 0.00001) and FINS (I^2^ = 91%, P < 0.00001). The heterogeneities justified the use of random-effects model in meta-analysis.

**Table 2:** Results of overall efficacy

**Outcome**	**No. of studies**	**Pooled sample size**	**Heterogeneity**			**Overall effect**	
			**T**^**2**^	**I**^**2**^	**P-value**	**SMD [95% CI]**	**P-value**
BMI	10	988	0.50	92%	<0.00001	-0.06 [-0.53, 0.40]	0.79
SBP	5	615	0.22	86%	<0.0001	0.39 [-0.06, 0.83]	0.09
DBP	5	615	0.23	87%	<0.00001	0.34 [-0.12, 0.79]	0.15
FPG	14	1611	0.03	47%	0.03	-0.02 [-0.16, 0.12]	0.80
HbA1c	13	1543	0.02	41%	0.06	0.01 [-0.13, 0.14]	0.91
PPBS	11	1099	0.18	81%	<0.00001	-0.27 [-0.56, 0.01]	0.06
TC	9	887	0.16	79%	<0.0001	0.33 [0.03, 0.63]	0.03
HDL	9	887	0.24	85%	<0.00001	0.11 [-0.25, 0.46]	0.56
LDL	6	702	0.02	29%	0.21	0.35 [0.16, 0.53]	0.0002
TG	9	887	0.05	54%	0.03	0.26 [0.05, 0.46]	0.01
FINS	10	1019	0.41	91%	<0.00001	0.07 [-0.35, 0.50]	0.73

### Risk of bias across studies

**Figure 2:**
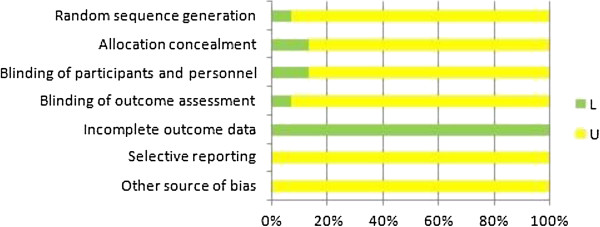
Cochrane’s risk of bias.

Cochrane’s risk of bias tool was used to assess the RCT quality (Table 3 and Figure 2). The attrition bias of all included studies was low (few missing data). Other key aspects among studies were mostly unclear in risk of bias except two studies [20,36].

**Table 3:** Cochrane’s risk of bias

**Source of bias**	**Random sequence generation**	**Allocation concealment**	**Blinding of participants and personnel**	**Blinding of outcome assessment**	**Incomplete outcome data**	**Selective reporting**	**Other source of bias**
Ling (2003) [33]	U	U	U	U	L	U	U
Ramachandran (2004) [34]	U	U	U	U	L	U	U
Derosa (2004) [35]	U	U	U	U	L	U	U
Gonzolez (2004) [36]	U	L	L	L	L	U	U
Rong (2004) [22]	U	U	U	U	L	U	U
Tang (2004) [37]	U	U	U	U	L	U	U
Yamanouchi (2005) [20]	L	L	L	U	L	U	U
Ning (2006) [38]	U	U	U	U	L	U	U
Wu (2007) [39]	U	U	U	U	L	U	U
Xu (2007) [40]	U	U	U	U	L	U	U
Li (2007) [41]	U	U	U	U	L	U	U
Wang (2009) [21]	U	U	U	U	L	U	U
Rahman (2011) [42]	U	U	U	U	L	U	U
Yoon (2011) [21]	U	U	U	U	L	U	U
Wang (2011) [43]	U	U	U	U	L	U	U

Note: L, low risk of bias; U, unclear risk of bias; H, high risk of bias.

### Sensitivity analysis

Sensitivity analysis checked whether the overall effects would be different if only the studies with the sample size N ≥ 90 were included. As shown in Table 4, metformin outperformed glimepiride only on LDL (0.41 [0.21, 0.61], P < 0.0001) in the studies with sample size N ≥ 90. Other outcomes such as FPG, BMI, TC and TG did not show significant difference between glimepiride and metformin.

**Table 4:** Sensitivity analysis (sample size ≥ 90)

**Outcome**	**No. of studies**	**Pooled sample size**	**Heterogeneity**			**Overall effect**	
			**T**^**2**^	**I**^**2**^	**P-value**	**SMD [95% CI]**	**P-value**
BMI	4	605	0.05	65%	0.03	0.21 [-0.06, 0.49]	0.13
FPG	8	1228	0.01	30%	0.18	0.09 [-0.04, 0.23]	0.18
HbA1c	8	1228	0.03	56%	0.03	0.06 [-0.12, 0.23]	0.52
PPBS	6	792	0.00	0%	0.59	-0.01 [-0.15, 0.13]	0.85
TC	3	504	0.22	90%	<0.0001	0.56 [0.00, 1.12]	0.05
HDL	3	504	0.18	88%	0.0002	-0.27 [-0.78, 0.24]	0.30
LDL	3	504	0.01	21%	0.28	0.41 [0.21, 0.61]	<0.0001
TG	3	504	0.00	0%	0.61	0.13 [-0.05, 0.30]	0.16
FINS	5	698	0.44	93%	<0.00001	0.37 [-0.23, 0.98]	0.22

Sensitivity analysis also considered whether prior use of anti-diabetic drugs would affect the RCT results. Table 5 shows that only the effect of metformin on BMI became statistically significant after excluding the studies with participants who were non-responders to other sulfonyureas. The significance of the effects of metformin on TC, LDL, TG and other aspects remained the same, indicating the overall results on those aspects were robust.

**Table 5:** Sensitivity analysis based on drug treatment before trials

**Criteria**	**Outcome**	**No. of studies**	**Pooled sample size**	**Heterogeneity**			**Overall effect**	
				**T**^**2**^	**I**^**2**^	**P-value**	**SMD [95% CI]**	**P-value**
Excluding the studies with non-responders to other sulfonylureas	BMI	10	988	0.50	92%	<0.00001	-0.06 [-0.53, 0.40]	0.79
	SBP	5	615	0.22	86%	<0.0001	0.39 [-0.06, 0.83]	0.09
	DBP	5	615	0.23	87%	<0.00001	0.34 [-0.12, 0.79]	0.15
	FPG	12	1314	0.04	48%	0.03	0.01 [-0.15, 0.17]	0.91
	HbA1c	11	1246	0.03	46%	0.05	0.04 [-0.12, 0.19]	0.65
	PPBS	9	802	0.27	85%	<0.00001	-0.32 [-0.69, 0.06]	0.10
	TC	9	887	0.16	79%	<0.0001	0.33 [0.03, 0.63]	0.03
	HDL	9	887	0.24	85%	<0.00001	0.11 [-0.25, 0.46]	0.56
	LDL	6	702	0.02	29%	0.21	0.35 [0.16, 0.53]	0.0002
	TG	9	887	0.05	54%	0.03	0.26 [0.05, 0.46]	0.01
	FINS	8	722	0.58	92%	<0.00001	0.11 [-0.44, 0.67]	0.69
Excluding the studies with participants who used other anti-diabetic drugs before RCTs	BMI	6	687	0.05	56%	0.04	0.27 [0.03, 0.51]	0.03
	SBP	3	444	0.22	87%	0.0004	0.60 [0.03, 1.16]	0.04
	DBP	3	444	0.29	90%	<0.0001	0.52 [-0.12, 1.16]	0.11
	FPG	8	989	0.07	66%	0.004	-0.03 [-0.26, 0.19]	0.78
	HbA1c	7	921	0.05	61%	0.02	-0.00 [-0.22, 0.22]	1.00
	PPBS	5	477	0.54	92%	<0.00001	-0.60 [-1.28, 0.08]	0.08
	TC	6	687	0.18	82%	<0.0001	0.38 [0.00, 0.76]	0.05
	HDL	6	687	0.12	76%	0.0009	-0.14 [-0.46, 0.19]	0.41
	LDL	4	572	0.04	56%	0.08	0.30 [0.05, 0.56]	0.02
	TG	6	687	0.00	0%	0.74	0.13 [-0.02, 0.28]	0.10
	FINS	5	483	0.53	92%	<0.00001	0.43 [-0.24, 1.10]	0.21

Sensitivity analysis tested whether the efficiency of metformin would be different if the daily dose of metformin was less than 1000 mg. Table 6 shows that metformin outperformed glimepiride only on BMI (0.33 [0.09, 0.58], P = 0.007) with a low daily dose and on LDL (0.43 [0.12, 0.74], P = 0.006) with a relative high daily dose.

**Table 6:** Sensitivity analysis based on the daily doses of metformin

**Daily doses**	**Outcome**	**No. of studies**	**Pooled sample size**	**Heterogeneity**			**Overall effect**	
				**T**^**2**^	**I**^**2**^	**P-value**	**SMD [95% CI]**	**P-value**
Below 1000 mg only	BMI	4	352	0.01	22%	0.28	0.33 [0.09, 0.58]	0.007
	SBP	2	177	0.00	0%	0.64	-0.02 [-0.31, 0.28]	0.92
	DBP	2	177	0.00	0%	0.67	-0.06 [-0.36, 0.23]	0.69
	FPG	5	584	0.02	34%	0.19	0.03 [-0.18, 0.24]	0.77
	HbA1c	5	584	0.07	65%	0.02	0.14 [-0.15, 0.44]	0.34
	PPBS	3	276	0.04	46%	0.16	0.02 [-0.32, 0.36]	0.91
	TC	3	251	0.04	41%	0.18	0.32 [-0.03, 0.66]	0.07
	HDL	3	251	0.00	0%	0.83	0.01 [-0.23, 0.26]	0.91
	LDL	1	136	NA	NA	NA	0.19 [-0.15, 0.53]	0.27
	TG	3	251	0.00	0%	0.67	0.10 [-0.15, 0.34]	0.44
	FINS	4	352	0.00	4%	0.37	0.22 [0.00, 0.43]	0.05
1000 mg and above 1000 mg	BMI	6	636	0.87	95%	<0.00001	-0.36 [-1.13, 0.41]	0.36
	SBP	3	438	0.17	85%	0.002	0.64 [0.12, 1.16]	0.02
	DBP	3	438	0.19	86%	0.0009	0.60 [0.06, 1.14]	0.03
	FPG	9	1027	0.04	55%	0.02	-0.05 [-0.24, 0.14]	0.64
	HbA1c	8	959	0.00	0%	0.87	-0.08 [-0.21, 0.04]	0.20
	PPBS	8	823	0.22	84%	<0.00001	-0.37 [-0.73, -0.01]	0.04
	TC	6	636	0.24	85%	<0.00001	0.34 [-0.09, 0.77]	0.12
	HDL	6	636	0.39	90%	<0.00001	0.14 [-0.39, 0.68]	0.59
	LDL	5	566	0.02	32%	0.21	0.38 [0.17, 0.59]	0.0004
	TG	6	636	.0.08	67%	0.010	0.36 [0.07, 0.64]	0.02
	FINS	6	667	0.71	95%	<0.00001	-0.02 [-0.72, 0.67]	0.94
Above 1000 mg only	BMI	2	234	0.11	72%	0.06	0.16 [-0.38, 0.69]	0.56
	SBP	2	234	0.42	91%	0.001	0.69 [-0.25, 1.63]	0.15
	DBP	2	234	0.47	92%	0.0005	0.63 [-0.36, 1.63]	0.21
	FPG	3	461	0.07	73%	0.02	0.02 [-0.35, 0.38]	0.93
	HbA1c	3	461	0.00	0%	0.91	-0.16 [-0.34, 0.03]	0.10
	PPBS	3	461	0.00	0%	0.82	-0.11 [-0.29, 0.07]	0.24
	TC	2	234	0.01	17%	0.27	0.23 [-0.07, 0.52]	0.13
	HDL	2	234	0.00	0%	0.48	0.06 [-0.20, 0.32]	0.64
	LDL	1	164	NA	NA	NA	0.43 [0.12, 0.74]	0.006
	TG	2	234	0.04	49%	0.16	0.16 [-0.23, 0.55]	0.42
	FINS	4	531	0.71	95%	<0.00001	0.28 [-0.57, 1.13]	0.52

Note: "NA" stands for "not applicable".

### Subgroup analysis

Meta-analysis of the subgroups with different follow-up periods (12-24 weeks and 48-60 weeks) showed that metformin moderated BMI and TC better than glimepiride in the shorter term while both drugs were equivalent in performance in all aspects except LDL in the longer term. As shown in Table 7, metformin performed better than glimepiride on both BMI (0.47 [0.24, 0.69], P < 0.0001) and TC (0.50 [0.27, 0.72], P < 0.0001) in 12-24 weeks subgroup. In 48-60 weeks subgroup, metformin performed better only on LDL (0.48 [0.29, 0.67], P < 0.00001).

**Table 7:** Subgroup analysis of different follow-up periods

**Follow-up period**	**Outcome**	**No. of studies**	**Pooled sample size**	**Heterogeneity**			**Overall effect**	
				**T**^**2**^	**I**^**2**^	**P-value**	**SMD [95% CI]**	**P-value**
12-24 weeks	BMI	4	307	0.00	0%	0.69	0.47 [0.24, 0.69]	<0.0001
	FPG	7	698	0.03	39%	0.13	-0.08 [-0.27, 0.12]	0.46
	HbA1c	7	698	0.07	61%	0.02	-0.04 [-0.29, 0.20]	0.72
	PPBS	2	297	0.00	0%	0.78	-0.15 [-0.38, 0.08]	0.20
	TC	4	307	0.00	0%	0.98	0.50 [0.27, 0.72]	<0.0001
	HDL	4	307	0.00	0%	0.66	0.12 [-0.10, 0.35]	0.28
	LDL	2	198	0.00	0%	0.36	0.28 [-0.00, 0.56]	0.05
	TG	4	307	0.00	0%	0.65	0.21 [-0.01, 0.44]	0.07
	FINS	5	542	0.05	54%	0.07	0.05 [-0.21, 0.32]	0.69
48-60 weeks	BMI	5	613	0.84	96%	<0.00001	-0.63 [-1.46, 0.20]	0.14
	SBP	4	545	0.26	89%	<0.00001	0.43 [-0.10, 0.96]	0.11
	DBP	4	545	0.27	89%	<0.00001	0.39 [-0.15, 0.93]	0.16
	FPG	7	913	0.04	53%	0.05	0.03 [-0.17, 0.23]	0.77
	HbA1c	6	845	0.00	0%	0.43	0.03 [-0.10, 0.17]	0.65
	PPBS	3	333	0.00	0%	0.95	-0.02 [-0.23, 0.20]	0.88
	TC	4	512	0.37	91%	<0.00001	0.23 [-0.39, 0.86]	0.47
	HDL	4	512	0.53	94%	<0.00001	0.10 [-0.64, 0.84]	0.79
	LDL	3	436	0.00	0%	0.80	0.48 [0.29, 0.67]	<0.00001
	TG	4	512	0.14	80%	0.002	0.31 [-0.11, 0.73]	0.15
	FINS	4	409	1.16	96%	<0.00001	0.14 [-0.94, 1.21]	0.80

Meta-analysis of the subgroups with BMI below or above 27.5 (i.e. the norm in the countries where the included RCTs were conducted) [44] was also conducted. As shown in Table 8, metformin outperformed glimepiride on control of FPG (0.34 [0.11, 0.57], P = 0.003), TC (0.33 [0.08, 0.58], P = 0.01) and LDL (0.32 [0.08, 0.56], P = 0.008) in the higher BMI subgroup (BMI ≥ 27.5). However, in the lower BMI subgroup (BMI < 27.5) metformin and glimepiride were not significantly different in performance as determined by outcome measures.

**Table 8:** Subgroup analysis of different BMI

**BMI**	**Outcome**	**No. of studies**	**Pooled sample size**	**Heterogeneity**			**Overall effect**	
				**T**^**2**^	**I**^**2**^	**P-value**	**SMD [95% CI]**	**P-value**
BMI < 27	BMI	7	618	0.83	94%	<0.00001	-0.27 [-0.98, 0.43]	0.45
	SBP	3	381	0.09	72%	0.03	0.20 [-0.21, 0.60]	0.34
	DBP	3	381	0.10	74%	0.02	0.15 [-0.27, 0.57]	0.48
	FPG	7	788	0.02	38%	0.14	-0.05 [-0.24, 0.13]	0.57
	HbA1c	6	720	0.00	0%	0.43	0.06 [-0.09, 0.20]	0.43
	PPBS	4	276	0.23	780%	0.003	-0.38 [-0.91, 0.15]	0.16
	TC	5	455	0.37	88%	<0.00001	0.26 [-0.31, 0.84]	0.37
	HDL	5	455	0.57	92%	<0.00001	0.11 [-0.58, 0.81]	0.75
	LDL	3	340	0.08	65%	0.06	0.30 [-0.09, 0.70]	0.14
	TG	5	455	0.13	72%	0.006	0.33 [-0.05, 0.71]	0.09
	FINS	5	352	0.21	78%	0.001	-0.17 [-0.63, 0.29]	0.47
BMI ≥ 27	BMI	3	370	0.13	79%	0.009	0.31 [-0.15, 0.78]	0.18
	SBP	2	234	0.42	91%	0.001	0.69 [-0.25, 1.63]	0.15
	DBP	2	234	0.47	92%	0.0005	0.63 [-0.36, 1.63]	0.21
	FPG	2	300	0.00	0%	0.82	0.34 [0.11, 0.57]	0.003
	HbA1c	2	300	0.27	91%	0.001	0.17 [-0.58, 0.93]	0.65
	PPBS	2	300	0.01	29%	0.24	0.09 [-0.18, 0.36]	0.51
	TC	3	370	0.02	30%	0.24	0.33 [0.08, 0.58]	0.01
	HDL	3	370	0.00	0%	0.71	0.03 [-0.18, 0.23]	0.79
	LDL	2	300	0.00	6%	0.30	0.32 [0.08, 0.56]	0.008
	TG	3	370	0.00	4%	0.35	0.15 [-0.06, 0.36]	0.17
	FINS	3	370	0.76	95%	<0.00001	0.56 [-0.45, 1.57]	0.28

### Adverse events

Eight out of 15 studies reported adverse events. As shown in Table 9, glimepiride had more hypoglycemia episodes than metformin did (4.94 [2.03, 11.99], P = 0.0004). Incidents of gastrointestinal upset, including diarrhea, epigastric discomfort, stomach pain and abdominal distension, were reported more frequently in metformin group (0.07 [0.01, 0.37], P = 0.002). Overall, these two drugs had no significant difference in side effects (0.35 [0.06, 2.01], P = 0.24) among the included RCTs.

**Table 9:** Adverse events

**Adverse events**	**No. of studies**	**Pooled sample size**	**Heterogeneity**			**Overall effect**	
			**T**^**2**^	**I**^**2**^	**P-value**	**OR [95% CI]**	**P-value**
All side effects	8	1003	4.70	81%	<0.00001	0.35 [0.06, 2.01]	0.24
Hypoglycemia	5	542	0.00	0%	0.77	4.94 [2.03, 11.99]	0.0004
Gastrointestinal upset	5	763	2.27	61%	0.04	0.07 [0.01, 0.37]	0.002

### Publication bias

**Figure 3:**
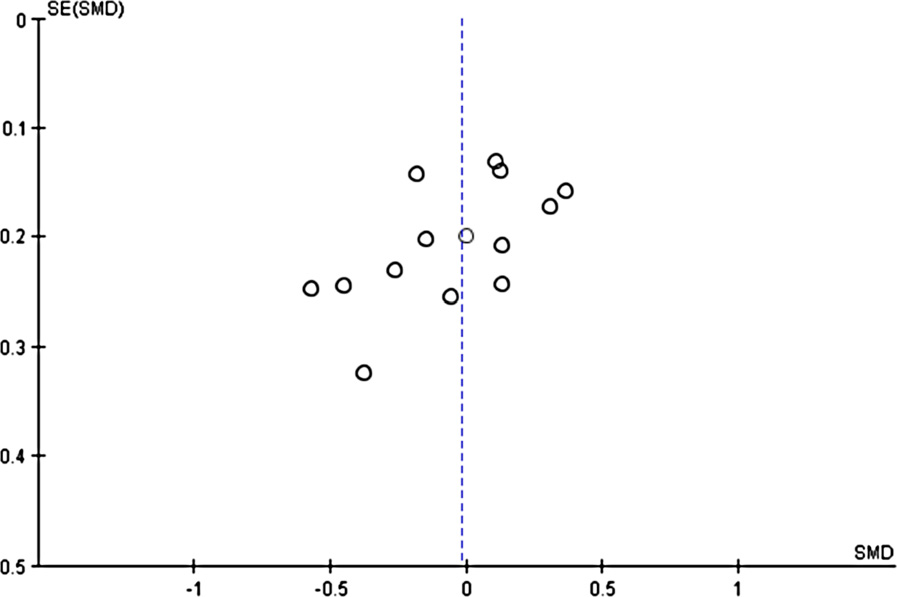
Funnel plots of publication biases in FPG.

Funnel plots were generated to visualize possible publication bias. Major outcomes FPG, BMI, HbA1c, PPBS, TC, FINS, HDL, LDL, TG, SBP and DBP showed moderate asymmetries across studies in the funnel plots, indicating there was publication bias. A typical funnel plot is shown in Figure 3. The statistical significance of the moderate publication bias in sugar (FPG and PPBS) and lipid (HDL and TG) indices was confirmed by the Begg’s rank correlation test [45]. Egger’s linear regression method [46] further confirmed the statistical significance of the publication bias in FPG towards metformin (Table 10), indicating that the difference in glycemic control efficacy between metformin and glimepiride was less than it seemed.

**Table 10:** Results of Begg and Egger's tests

**Outcome**	**No. of studies**	**Begg’s test**		**Egger’s test**	
		**Z**	**P-value**	**Kendall’s tau**	**P-value**
BMI	10	-2.9174	0.0035	-0.0222	1.0000
SBP	5	-1.3229	0.1859	-0.2000	0.1867
DBP	5	-1.5941	0.1109	-0.4000	0.4833
FPG	14	-2.2496	0.0245	-0.4286	0.0356
HbA1c	13	-0.9014	0.3673	-0.2308	0.3062
PPBS	11	-2.2267	0.0260	-0.4182	0.0866
TC	9	-0.8218	0.4112	-0.0556	0.9195
HDL	9	2.2070	0.0273	0.5000	0.0752
LDL	6	-1.1076	0.2680	-0.3333	0.4694
TG	9	0.8232	0.04104	0.2778	0.3585
FINS	10	-0.8661	0.3864	-0.2444	0.3807

## Discussion

Metformin remains the most effective monotherapy of T2DM while its advantages are diminishing among newer anti-diabetic drugs. Past studies comparing the efficacy between metformin with sulfonylureas showed that metformin was significantly better in controlling HbA1c, FPG, BMI, LDL and TG [3-5,7]. Glimepiride is a better sulphonylurea in treating T2DM [17-19,47,48]. The present meta-analytic study found that metformin was not significantly better than glimepiride, particularly in controlling HbA1c, FPG and BMI.

This meta-analysis supported that both metformin and glimepiride was effective in treating T2DM for glycemic control. Metformin performed better than glimepiride in management of BMI and lipid metabolism indices but the advantages of metformin were only significant in short follow-up periods.

These results were deemed robust after evaluation by sensitivity analysis that excluded small RCTs and the participants who were non-responders to non-glimepiride sulfonylureas or who received anti-diabetic treatment. The differences between metformin and glimepiride became insignificant in large RCTs. Even for treating the patients who were not responding to conventional (non-glimepiride) sulfonylureas, glimepiride and metformin were equivalent in glycemic control. This finding could not be achieved by comparing sulfonylureas (including glimepiride) as a group with metformin.

Adverse events analysis showed that glimepiride had more hypoglycemia episodes, in agreement with previous results that metformin was associated with less hypoglycemia than sulphonylureas [3] and that metformin had a higher risk of gastrointestinal upset [49-51]. The weight gain side effects were not significant in the included RCTs, in consistency with the findings of previous studies [52] on the weight gain issue of glimepiride.

The daily dose of metformin affected the efficacy and side effects of metformin. When the daily dose of metformin was more than 1000 mg, the probability of gastrointestinal upset would be increased exponentially [53]. Sensitivity analysis on the daily doses of metformin showed that metformin outperformed glimepiride only on BMI with a low daily dose and on LDL with a relatively high daily dose, but was not significantly better than glimepiride on controlling HbA1c, FPG and Fins in all daily doses.

Most of the included RCTs (13 out of 15 in this meta-analysis) were conducted in Asia; thus, the Asian norm of BMI for subgroup analysis was adopted. It seems that Asian patients were less affected by the weight gain side effect of glimepiride. Multi-country and multi-ethnic trials are warranted to test whether glimepiride is more suitable for Asian patients. Double-blind RCTs with longer follow-up periods should be conducted to assess other side effects such as those on cardiovascular system. It is encouraging that new clinical trials comparing metformin and glimepiride for monotherapy of T2DM have been registered [54]. Hence, proper updates on this meta-analysis will be conducted in forthcoming years.

These results provide direct evidence to support ADA’s and NICE’s recommendations to consider glimepiride as one of the alternatives to metformin. Our meta-analysis of the RCT results demonstrated that the advantages of metformin over glimepiride were not always significant particularly in Asian patients.

## Conclusion

Metformin and glimepiride were not significantly different in glycemic control of T2DM, suggesting that glimepiride would be a good choice second to metformin in the monotherapy of T2DM.

## Abbreviations

BMI: Body mass index; DBP: Diastolic blood pressure; FPG: Fasting plasma glucose; FINS: Fasting plasma insulin; HbA1c: Glycosylated hemoglobin level; HDL: High-density lipoprotein; LDL: Low-density lipoprotein; PPBS: Postprandial blood sugar; RCT: Random control trials; SBP: Systolic blood pressure; TC: Total cholesterol; TG: Triglycerides; T2DM: Type 2 diabetes mellitus.

## Competing interests

The authors declare that they have no competing interests.

## Authors’ contributions

SL supervised this study. HZ and XZ extracted and analyzed the data from the selected studies according to the eligibility criteria. SZ repeated data analysis for cross-checking. Other authors assisted in searching the databases, assessing and selecting studies. HZ, SZ and SL interpreted the data and drafted a report on the findings. SL revised the manuscript for submission. All authors read and approved the final version of the manuscript.
